# Toe Macrodactyly, Macrodystrophia Lipomatosa, Fibrolipomatous Hamartoma and Lipomatosis of Nerve. Are they similar?: A Case Report

**DOI:** 10.5704/MOJ.2207.019

**Published:** 2022-07

**Authors:** A Syed, S Das, AH Abdul-Rashid, WSE Wan-Ahmad-Kamal, K Jamil

**Affiliations:** 1 Department of Orthopaedic and Traumatology, Universiti Kebangsaan Malaysia, Kuala Lumpur, Malaysia; 2 Department of Human and Clinical Anatomy, Sultan Qaboos University, Muscat, Oman; 3 Department of Diagnostic Laboratory Services, Universiti Kebangsaan Malaysia, Kuala Lumpur, Malaysia

**Keywords:** macrodactyly, anatomical dissection, macrodystrophia lipomatosa, lipomatosis of nerve, fibrolipomatous hamartoma

## Abstract

Macrodystrophia Lipomatosa (MDL) of the toe is a rare, congenital, disproportionate overgrowth involving one or more digits in the lower limb. Despite being a benign condition, when left untreated, it may cause physical impairment and interfere with daily activities. This form of localised gigantism is the result of excessive proliferation of fibroadipose tissue within the nerve along with associated macrodactyly. The mainstay of treatment is debulking or amputation to accommodate the patient’s daily activities, as well as for cosmesis. In this case report, the clinical and radiographic findings, anatomical descriptions, and histopathological findings are presented. The difference between MDL, fibrolipomatous hamartoma (FLH) and lipomatosis of the nerve (LON) are also discussed.

## Introduction

Macrodystrophia Lipomatosa (MDL) is a rare, congenital disorder causing focal overgrowth of mesenchymal elements, predominantly fibroadipose tissue. The term “Macrodystrophia Lipomatosa” was first coined by Feriz in 1925 to describe localised gigantism involving the lower limb^1^. MDL can occur with or without fibrolipomatous hamartoma (FLH) of the nerve which is characterised by expansion of the epineurium by adipose and fibrous tissue. It usually involves the peripheral nerves, and their accompanying branches with increased fatty tissue interspersed among thickened nerve bundles as well as perineural fibrosis. MDL and FLH of the nerve are described in literature as different pathologies despite being classified as the same entities in the latest WHO classification of Tumours of Soft Tissue and Bone^2^.

Due to its rarity and multiple terms used to describe the disorder, the exact incidence is unknown. Previous studies have described MDL involving the hands and foot with gigantism of the digits following the distribution pattern of the median nerve and medial plantar nerve, respectively^3^. However, to the best of our knowledge, to date, no published paper has given a detailed anatomical description of the MDL.

## Case Report

**Fig. 1:**
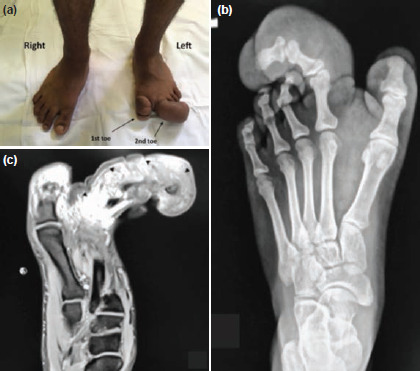
Clinical and radiological features of MDL (a) Clinical features of overgrowth of left 1st and 2nd toe. (b) Radiograph of MDL showing a laterally deviated 2nd toe with enlarged phalanges and soft tissue overgrowth. (c) Sagittal view of T1 weighted MRI of the foot showing excess subcutaneous tissue of the 2nd toe (arrowhead).

We report a case of a 15-year-old boy, who presented with enlarged first and second toes since birth. The deformities were painless and grew in size proportionate to him. However, due to difficulty in shoewear, he sought our expertise. Examination showed enlarged first and second left toes with lateral deviation (Fig. 1a). The radiograph revealed enlarged phalanges with the middle and distal phalanges deviating towards the third toe (Fig. 1b). Magnetic resonance imaging (MRI) showed thickened subcutaneous tissue surrounding the enlarged bony structures (Fig. 1c). Options of treatment were discussed with the patient which included debulking as well as amputation. The patient then opted for Ray amputation of the 2nd toe and the amputated specimen was examined. His review in the clinic six months after the surgery showed no recurrence of the growth. He is continued on a yearly follow-up to detect any recurrence. As the big toe did not cause any difficulties in shoewear, no surgical intervention was needed nor desired by the patient.

**Fig. 2:**
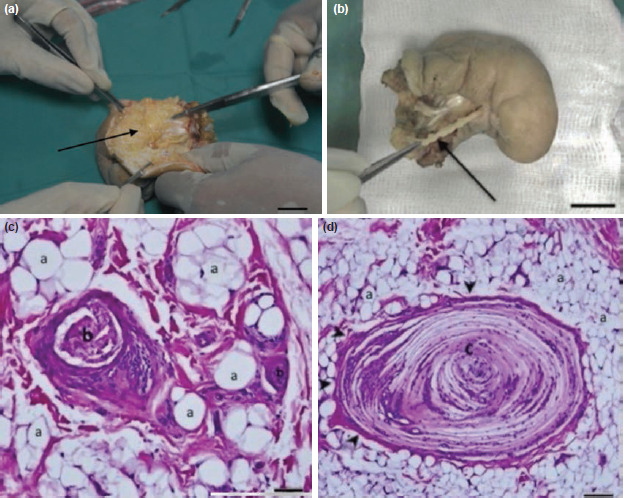
Anatomical dissection and histological examination. (a) Anatomical dissection of the left 2nd toe showing excess subcutaneous tissue (arrow) deep to the skin. Bar = 2cm. (b) Enlarged nerve (arrow) accompanying the overgrown toe. Bar = 2cm. (c) Histological examination showed expansion of the epineurium and perineurium by adipose tissue and separation of individual nerve bundles. a = adipose tissue, b = nerve bundle (H&E stain, Bar = 1mm at x40 magnification). (d) There is concentric perineural fibrosis (arrowhead) and pseudo-onion bulb formation. a = adipose tissue, c = nerve fascicle (H&E stain, Bar = 1 mm at x40 magnification).

Anatomical dissection of the excised specimen showed thick layers of fat deep to the reflected skin (Fig. 2a). The branches of the medial plantar nerve innervating the second toe were thickened (Fig. 2b) with enlarged middle and distal phalanges. No other abnormalities of other anatomical structures were noted.

Histological examination showed infiltration of the epineurium and perineurium by adipose and fibrous tissue (Fig. 2c), which dissected between and separated individual nerve bundles, and was associated with concentric perineural fibrosis and pseudo-onion bulb formation (Fig. 2d). These findings were consistent with the diagnosis of lipomatosis of the nerve.

## Discussion

MDL is a rare, non-hereditary congenital disorder with an undetermined aetiology. The proposed mechanisms include disturbed foetal circulation, errors in segmentation, trophic influence of nerve, and in utero disturbance of growth-limiting factor^4^.

Clinically, MDL has to be differentiated from other conditions such as plexiform neurofibromatosis, and Proteus syndrome. In this patient, the key defining clinical feature to suggest MDL was its presence at birth and the mass grew proportionately.

Prabhu *_et al_* classified MDL into three types according to the pattern of distribution^5^. One pattern observed was “nerve territory orientated type” with or without nerve enlargement. In their study, this is the most commonly observed pattern whereby soft tissues are hypertrophied by fat which may or may not infiltrate nerve^5^. The other patterns were “Diffuse lipomatous type” and “mixed pattern type”. In diffuse lipomatous type, there is involvement of the entire extremity with sparing of the nerves. The “mixed pattern type” is an overlap of “nerve territory-oriented type” and “diffuse lipomatous type”. It was however acknowledged by the author that the latter two patterns were only observed in three patients^5^. This form of classification provides clarity for authors when describing MDL with its various forms.

The radiological examination provides further insight when investigating MDL^3^. Plain radiographs may show an increase in width, length and cortical thickening of the phalanges with soft tissue overgrowth surrounding the osseous structures^5^. Ultrasound may show LON with fascicles and interfascicular fat hypertrophy^5^. Another role of an ultrasound is to exclude other disorders that may present with an overgrowth such as vascular malformations.

On MRI, the proliferation of mature adipocytes within peripheral nerves with perineural fibrosis is commonly used to describe FLH or LON^3^. The presence of nerve involvement can also be confirmed when there is an enlargement of the nerve with a coaxial cable appearance due to splaying the nerve fascicles by interfascicular adipose tissue^5^. Furthermore, MRI is able to exclude other pathologies such as neurofibromas or lymphangiomas. In our patient, MRI was able to detect enlarged phalanges as well as thickening of the subcutaneous layer. Nerve involvement was not obvious on the MRI until anatomical dissection was performed revealing extensive infiltration of the nerve by fibroadipose tissue.

Histologically, LON is characterised by the expansion of the epineurium by adipose and fibrous tissue. This condition has several synonyms, which include MDL, neural fibrolipoma and perineural lipoma^2^. Some authors believe lipomatosis of the nerve to be a hamartomatous lesion, thus it is also frequently known as FLH. Histological examination of an FLH lesion would show mature fibroadipose tissue infiltrating the nerve splaying the nerve fascicles causing expansion of the epineurium. The resulting onion bulb-like appearance is due to the increased perineural cells and perineural fibrosis.

Histological examination of the overgrowth remains the gold standard to diagnose MDL^3^. Then again, MDL, LON and FLH of the nerve are used interchangeably as they define the same histological and radiological findings with similar outcomes and prognosis. Nonetheless, for this case in the presence of macrodactyly, it may be more appropriate to use the term MDL.

MDL is an extremely rare condition that can present with enlargement of toes or digits. It may present with or without lipomatosis of the nerve. However, in the existing literature, MDL, FLH and LON are used interchangeably and this leads to confusion. We conclude that the term MDL, FLH of nerve or LON can be used in cases when there is nerve enlargement without subcutaneous, inter-muscular and intra-muscular fibrofatty hypertrophy. Non-invasive investigations such as ultrasound, radiographs or MRI may help establish a diagnosis and exclude other pathologies, but histological examination remains the gold standard for confirmation of diagnosis.
